# Potential Retinal Biomarkers in Alzheimer’s Disease

**DOI:** 10.3390/ijms242115834

**Published:** 2023-10-31

**Authors:** Mariana Yolotzin García-Bermúdez, Rupali Vohra, Kristine Freude, Peter van Wijngaarden, Keith Martin, Maj Schneider Thomsen, Blanca Irene Aldana, Miriam Kolko

**Affiliations:** 1 Eye Translational Research Unit, Department of Drug Design and Pharmacology, University of Copenhagen, 2100 Copenhagen, Denmark; 2 Department of Ophthalmology, Copenhagen University Hospital, Rigshospitalet, 2600 Glostrup, Denmark; 3 Group of Stem Cell Models and Embryology, Department of Veterinary and Animal Sciences, University of Copenhagen, 1870 Frederiksberg, Denmark; 4 Center for Eye Research Australia, Royal Victorian Eye and Ear Hospital, East Melbourne, VIC 3002, Australia; 5 Ophthalmology, Department of Surgery, University of Melbourne, Melbourne, VIC 3010, Australia; 6 Department of Clinical Neurosciences, University of Cambridge, Cambridge CB2 0QQ, UK; 7 Neurobiology Research and Drug Delivery, Department of Health, Science and Technology, Aalborg University, 9220 Aalborg, Denmark; 8 Neurometabolism Research Group, Department of Drug Design and Pharmacology, University of Copenhagen, 2100 Copenhagen, Denmark

**Keywords:** Alzheimer’s disease, retinal alterations, ocular biomarkers, mitochondria, metabolism

## Abstract

Alzheimer’s disease (AD) represents a major diagnostic challenge, as early detection is crucial for effective intervention. This review examines the diagnostic challenges facing current AD evaluations and explores the emerging field of retinal alterations as early indicators. Recognizing the potential of the retina as a noninvasive window to the brain, we emphasize the importance of identifying retinal biomarkers in the early stages of AD. However, the examination of AD is not without its challenges, as the similarities shared with other retinal diseases introduce complexity in the search for AD-specific markers. In this review, we address the relevance of using the retina for the early diagnosis of AD and the complex challenges associated with the search for AD-specific retinal biomarkers. We provide a comprehensive overview of the current landscape and highlight avenues for progress in AD diagnosis by retinal examination.

## 1. Introduction

Alzheimer’s disease (AD) is a neurodegenerative brain disorder and the most common cause of dementia [1,2]. Recently, AD has been recognized as a global public health priority by the World Health Organization [3]. It is characterized by the presence of extracellular amyloid-β (Aβ) plaques and intraneuronal neurofibrillary tangles (NFTs) consisting of hyperphosphorylated tau protein (pTau) [4,5].

The amyloid cascade hypothesis postulates that altered production and clearance of Aβ is the major driving factor for AD pathogenesis in the brain, as abnormal accumulation of Aβ disrupts synaptic function, leading to neuronal loss and the characteristic neurodegeneration [6,7]. It is also known that the blood-brain barrier is compromised in AD, allowing the extravasation of fibrinogen and inflammatory mediators that activate microglial cells [8,9]. Microglia act as resident phagocytes and mediate synapse pruning by engulfing synapses [10], and once activated, microglia tend to form clusters around Aβ plaques [11], which increases microglial proliferation [12]. In addition, microglial exosomes have been shown to propagate pTau [12,13], while the downregulation of microglial homeostatic genes involved in regulation has been reported to correlate with neuronal loss [14].

Activated microglia secrete inflammatory factors [10,15], including reactive oxygen species (ROS) [16]. Under normal physiological function, the reactive species generated can be controlled by antioxidant defenses [17]. However, in the early stages of AD, oxidative stress is present, and glia and neurons are highly sensitive to this [17], as brain cells respond to oxidative stress, they enter a cycle of increased ROS generation, causing more oxidative stress and mitochondrial damage, ultimately leading to cell death [18,19].

Screening for AD involves several techniques, including cognitive tests, neuroimaging, cerebrospinal fluid (CSF) analysis, blood tests, and genetic testing [20,21,22]. Cognitive tests assess memory and cognitive function, while neuroimaging techniques such as magnetic resonance imaging (MRI) and positron emission tomography (PET) can detect structural and protein changes in the brain [23]. CSF analysis and blood tests provide information on biomarkers associated with AD [21,24], and genetic testing can identify mutations in familial forms of the disease [25]. However, a definitive diagnosis can only be made at autopsy [26].

There are several difficulties with diagnosing AD. The most important is that early diagnosis remains challenging, as clinical symptoms often appear late in the disease [27]. In addition, the overlap of symptoms with other neurodegenerative diseases makes differentiation difficult [20], and biomarkers may lack specificity and be present in individuals without AD [28]. Accurate and widespread diagnosis is further complicated by access to specialized testing, ethical issues, and economic concerns. Despite these difficulties, research is underway to improve the accuracy and accessibility of AD detection. It also aims to find new ways to study the disease and detect it in its early stages.

Retinal alterations are one of the earliest signs of AD [29]. Several post-mortem studies have shown Aβ plaques and NFTs in the retinas of AD patients [29,30,31]. In parallel, the retinal glial cell types, such as microglia and macroglia, become activated and change their morphology and immune phenotype [32]. In particular, macroglia, namely Müller glia, and astrocytes, are important for retinal homeostasis [33]. Müller glia are the most abundant glial cell type in the retina [34] and require large amounts of energy to maintain their functions, such as ensuring the survival of retinal ganglion cells (RGCs) [19,35,36]. Increased levels of oxidative stress have been shown to decrease glutamate transport and to reduce both the glycolytic activity and the mitochondrial respiratory function in Müller glia [37]. As changes in glial cells and cellular energetics are implicated in both AD and retinal dysfunction, a growing body of research suggests that energetic instability in the retina may be a predictor of AD.

**Figure 1:**
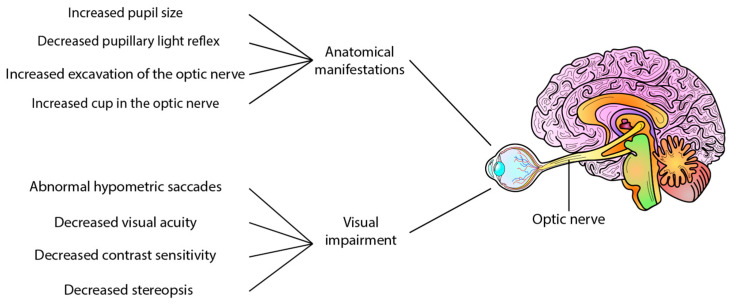
Visual changes in Alzheimer’s disease and erase AD. Several clinical and functional alterations have been reported in Alzheimer’s disease patients, such as decreased pupillary light reflex, increased pupil size, and increased excavation of the optic nerve. Other features include decreased contrast sensitivity, decreased visual acuity, stereopsis, abnormal hypometric saccade, and reduced visual sensitivity, among other alterations.

Refs. [38,39] Certain clinical signs have been observed in AD patients, including increased pupil size [40] and a less pronounced pupillary light reflex [41], and the presence of Aβ_40_ in the aqueous humour comparable to levels found in cerebrospinal fluid [42]. However, in post-mortem eyes of patients with AD, no deposits of Aβ have been reported in the lens [43]. Interestingly, increased cupping of the optic nerve has also been reported in AD [38], whereas a mouse study using Aβ injection found no axonal damage to the optic nerve [44]. In addition to anatomical manifestations, visual and motor impairments have also been reported in AD patients. These impairments include abnormal hypometric saccades, reduced visual acuity, impaired contrast sensitivity, stereopsis, lack of hand-eye coordination, impaired fixation of the eye, and difficulty in identifying objects [38,39,45] These changes provide predictive and progression information on neurodegenerative changes and represent potential ocular biomarkers of AD (Figure 1).

The diagnosis of AD is challenging because of the subjectivity of the methods used and the presence of other conditions that present with similar cognitive impairment, such as vascular dementia [46]. This results in limited sensitivity and specificity, leading to an inaccurate diagnosis in 10–15% of AD cases [47]. In recent years, considerable progress has been made in techniques for detecting AD-related disorders, but many of these are invasive and expensive, so a new approach to detecting AD, such as retinal screening, should be considered. At present, there are non-invasive and low-cost techniques for the detection of retinal and ocular changes that are associated with AD [48]. However, the similarity of the pathology to other diseases compels us to continue the study of changes that can be considered as specific retinal and ocular biomarkers in AD. This would also allow us to study AD from a new perspective, as the ocular and retinal changes already observed could be starting in the brain, allowing us to make a timely diagnosis and perhaps stop the progression of AD, giving patients a better quality of life.

In this review, we highlight the compelling parallels between the retinal and cerebral manifestations of AD given their close association. We provide a detailed account of the retinal structure and cell alterations observed in the context of AD. Furthermore, we offer insights into the intricate interplay between retinal cells and the resulting energetic impairment, oxidative stress, and mitochondrial dysfunction. At the same time, we outline the major challenges in retinal AD research caused by shared pathophysiological mechanisms with certain retinal diseases.

Lastly, we discuss the promising diagnostic techniques already available for the detection of retinal and ocular alterations in AD, which offer the dual advantage of early diagnosis and potentially dynamic monitoring of disease progression. This offers an alternative perspective for studying AD through retinal and ocular changes. However, it should be noted that some of these innovative approaches are still in the research phase with encouraging preliminary results.

## 2. The Retina: A Window to the Brain

The retina is a part of the central nervous system (CNS), originating from an out-pouching of the diencephalon [49]. The retina contains a high density of neurons that form a sensory extension of the brain [50]. Visual input is converted into neuronal signals in the photoreceptor cells and transmitted to the bipolar cells and then to the RGCs, the output neurons of the retina. RGC axons remain unmyelinated in the retina and converge on the optic nerve, where they become myelinated and travel to the visual centers in the brain. This lack of myelination in the intraretinal portions of RGC axons, which is essential for light to travel unimpeded to the photoreceptors, combined with a semi-hypoxic environment, renders the retina susceptible to metabolic stress [19].

The retina is dependent on a well-regulated process for the maintenance of a proper blood supply for the support of cellular functions [51]. Studies have shown changes in the retinal vasculature, the mid-peripheral retina [52], and the foveal avascular zone (FAZ) in AD, including changes in vessel density, thickness, and integrity [53,54]. Angiogenesis can be studied in a non-invasive way and since it is associated with AD [55], it is considered a possible diagnostic biomarker, but it requires further standardization.

### 2.1. Outer Retinal Changes in AD Patients

The outer retina, the outermost layer of cells in the retina, consists of photoreceptor cells and the retinal pigment epithelium (RPE) [56,57], which has received increasing attention in AD. However, compared to the inner retina, its role in AD is less well understood. The photoreceptor cells, which are essential for the capture of light and the initiation of the visual process, have shown signs of progressive degeneration in AD mice [58], potentially contributing to the visual impairment associated with the disease.

Optical coherence tomography (OCT) studies have correlated volumetric changes in the outer retina, ellipsoid zone to the retinal pigmented epithelium, with changes in brain volume in patients with AD, reflecting photoreceptor outer segment shortening [59]. Photoreceptor dysfunction accompanied by Aβ deposition is a common observation in animal models of AD [60,61]. In addition, a clinical study including 39 patients with mild AD and 21 patients with moderate AD showed impaired color perception in addition to photoreceptor degeneration compared to controls by OCT [62].

Visual impairment, including problems with contrast sensitivity, color discrimination, and depth perception, is a recognized symptom of AD [38,45]. Although the exact mechanisms remain unclear, these visual problems suggest a link between AD and the outer retina. Further research is needed to establish a definitive link and to better understand the role of the outer retina in AD.

### 2.2. Inner Retinal Alterations

The inner retina, which extends from the inner limiting membrane to the outer plexiform layer, consists of RGCs, the nerve fiber layer, and bipolar cells [63]. The RGCs are responsible for the transmission of visual information from the retina to the brain and show signs of degeneration in people with AD [38]. The thinning of the nerve fiber layer (NFL) and ganglion cell layer (GCL) in people with AD has been demonstrated using advanced imaging techniques such as OCT [38,64,65], suggesting structural changes in the inner retina.

In addition, post-mortem examinations of the retinas of patients with AD show the presence of Aβ in the NFL and GCL [31,66]. The presence of Aβ deposits, Aβ40 and Aβ42 alloforms, vascularization, and inflammation have been reported in the retinas of AD patients and mice [30,66,67,68].

Furthermore, pTau accumulation has been found in the inner plexiform layer and in the GCL where it has been linked to RGCs loss and inflammation [29,38,69]. Several post-mortem studies have reported an increase in pTau in the peripheral retina of people with AD [70]. This information highlights the link between AD-related changes in the brain and manifestations in the retina.

### 2.3. Microglia Abnormalities

Microglia are the resident immune cells of the CNS, including the brain and retina, where they are involved in the maintenance of homeostasis [71,72]. Microglia play a vital role in brain plasticity and development [73] through the removal of synapses from neuronal cell bodies [74,75]. Furthermore, microglia are important contributors to proper synaptic processing in health but are also involved in pathological conditions by disrupting neuronal connections [76]. Although astrocytes and microglia do not yet appear to play a direct role in synapse degeneration, neurons appear to be more active in this process [77].

Under normal circumstances, both the Triggering Receptor Expressed in Myeloid Cells 2 (TREM2) and Apolipoprotein E (ApoE) are involved in microglial cells in the phagocytosis of apoptotic neurons in the brain [78]. However, mutations in TREM2 are associated with an increased risk of late-onset AD as Aβ is not cleared properly, resulting in increased accumulation [10,79]. Increased levels of pro-inflammatory mediators such as interleukin-1β (IL-1β), IL-6, and tumor necrosis factor-alpha (TNF-α) have been found in the cerebrospinal fluid, contributing to neuroinflammation in AD [12,80]. Aβ activation of microglia requires the participation of the receptor complex CD36, α6β1 integrin, and CD47, which are involved in the internalization of Aβ [81], and receptors such as CD14 and Toll-like receptors 2 and 4 (TLR-2 and TLR-4) [82,83,84].

Under physiological conditions, microglia are found in the inner retina. As in the brain, they can be activated to remove debris and pathogens in response to injury or infection [71]. And in AD, activated microglia can infiltrate the outer retina [72].

### 2.4. Macroglia: The Importance of Müller Glia in AD

In addition to their homeostatic and metabolic support functions, Müller glia is also involved in the regulation of synaptic activity in the inner retina [15,85]. Müller glia are also sensitive to microglial changes [86] and can be activated in neurodegenerative diseases such as AD. Müller glia prevents the accumulation of glutamate in the synaptic clefts via excitatory amino acid transporters 1 and 2 (EAAT1 and EAAT2, also known as GLT-1), thereby preventing excitotoxicity [87]. Müller glia requires substantial bioenergetic support, provided primarily by a large number of mitochondria, to perform glutamate uptake and maintain retinal homeostasis [88].

The retina is one of the highest lactate-containing organs in the human body relative to size, and lactate has been shown to act as an energy substrate for retinal cells [34,89,90,91]. Müller glia and RGCs are the main cells responsible for lactate production in the retina [89]. Studies have shown increased cell survival in both cultures of isolated primary RGCs and Müller glia from neonatal mice (C57BL/6J) retinas when exposed to L-lactate. In addition to acting as an energy substrate, lactate also activates the G-coupled protein receptor 81 (GPR81) [34]. Lactate has been shown to have regulatory functions in the retina, such as increasing glutamate uptake, improving mitochondrial function and increasing glucose consumption, protecting against neuroinflammation, and providing neuroprotection in neurodegenerative conditions [89].

**Figure 2:**
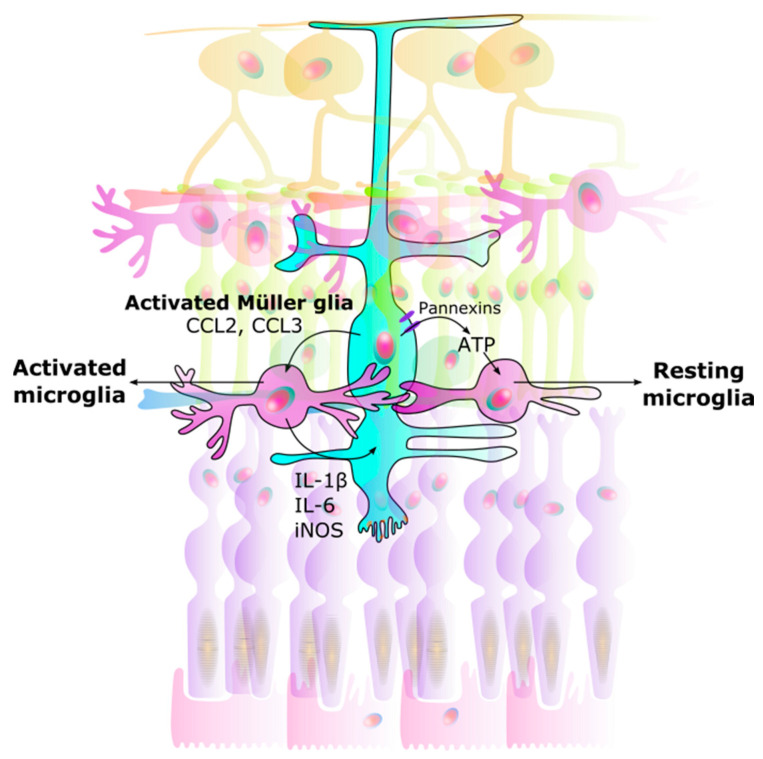
Interaction between Müller glia and microglia. Signals from Müller glia induce changes in microglia, causing them to either rest or become activated: (1) Activated microglia: Once activated, Müller glia activate microglia by CCL2 and CCL3 secretion. (2) Neuroinflammation: Microglia in turn produce IL-1β, IL-6, and iNOS in a positive feedback loop. This results in unregulated over-activation leading to neurodegeneration. (3) Resting microglia: ATP secretion through Müller glial pannexin channels allows microglia to rest. CCL Chemokine (C-C motif) ligand. IL Interleukin. iNOS Inducible nitric oxide synthase. ATP Adenosine triphosphate.

The bidirectional interaction between microglia and Müller glia is thought to act as a regulator in neuron-microglia interaction. Müller glia can detect neurotransmitter signals from neuronal and synaptic activity [86,87,92]. Adenosine triphosphate (ATP) is also known as a chemotactic agent [93], and Müller glia, via ATP secretion, can mediate activity dependent regulation of microglial dynamics [87,89], as well as regulate retinal blood flow [94]. The release of ATP from Müller glia through pannexin channels drives a resting state in microglia [95] (Figure 2).

## 3. Intricate Connection Means the Retina Mirrors the Brain

Findings from in vivo and in vitro studies in humans and animal models show the similarities in the manifestations of AD in the brain and retina. Post-mortem studies of the retina in humans and some animal models of AD have shown activation of microglia and Müller glia, as well as disturbances in energetic, metabolic, and mitochondrial processes. This suggests that Aβ accumulation triggers neurodegeneration in the retina similar to that observed in the brain [35,96] (Table 1).

Aβ accumulation in the brain has been found decades before the onset of symptoms [136,137], and several reports indicate that retinal Aβ follows a similar time course to that in the brain [67,138]. Retinal abnormalities have also been associated with AD progression and cognitive decline. These include loss of RGCs [120], reduced thickness of the retinal NFL (RNFL) and RGC layer and reduced retinal blood flow [102,121,139], as well as decreased thickness in the macular area as a result of inner layer degeneration [62]. Taken together, this suggests that the eye can be considered a window to study AD.

### 3.1. Energetic Impairment

**Figure 3:**
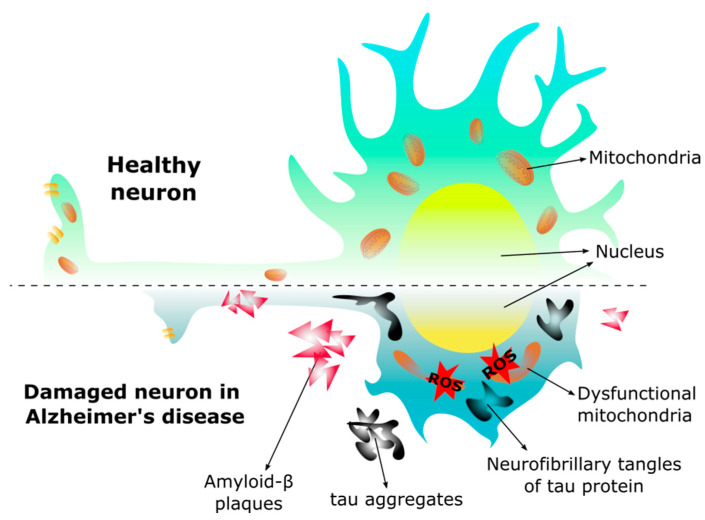
Morphology in the healthy neuron and the neuron of Alzheimer’s disease. In Alzheimer’s disease the neuron changes morphology, the dendrites are shorter, the axon is reduced decreasing the synapse, and the mitochondria become dysfunctional due to the presence of amyloid-β (Aβ) plaques and neurofibrillary tangles, leading to an increase of reactive oxygen species (ROS), and subsequent cell death.

The brain has a remarkably high energy requirement, with glucose being the main energy substrate [104]. However, lactate, ketone bodies, glycogen, and amino acids can also be used as bioenergetic substrates under certain circumstances [118]. Pathogenic factors such as abnormalities in glucose transport, impaired glucose metabolism, and mitochondrial dysfunction in the brain occur in AD and have been characterized in animal models [108,118,140] (Figure 3).

Like the brain, the retina is one of the most energy-demanding tissues in the body. To meet its vast energy demands [141], the retina is rich in mitochondria, particularly in the RPE, photoreceptors, and Müller glia, as well as in the RGC axons at the optic nerve head [102,142]. To achieve this, the structure and shape of the mitochondria are maintained by a balance of mitochondrial fission and fusion. Fission is the mitochondrial fragmentation that occurs when dynamin-related protein 1 (Drp1) and mitochondrial fission 1 protein (Fis1) are recruited to the outer mitochondrial membrane, resulting in smaller and more functional mitochondria [143]. Fusion allows mitochondria to support each other, compensating for defects in damaged mitochondria by physically fusing the outer and inner membranes of two different mitochondria [144]. This process also protects mitochondria from harmful DNA mutations and allows them to change shape for specific functions. The lack of mitochondrial fusion can therefore increase ROS production and decrease ATP synthesis [145]. Reports have suggested that alterations in these mitochondrial dynamics may be involved in neurodegenerative diseases. In AD, impaired fission-fusion dynamics can lead to mitochondrial dysfunction in the brain [146,147,148], with reduced levels of Drp1 and increased levels of Fis1 reported [149].

Deposition of Aβ has been shown to affect mitochondrial function. For instance, even mild increases in Aβ have been found to have detrimental effects on mitochondria [150]. In addition, several studies have indicated that mitochondrial morphology and function can be affected by inhibition of protein import [151], by the interaction of Aβ with the cyclophilin D-dependent mitochondrial permeability transition pore (mPTP) [152,153], and by its binding to mitochondrial Aβ-binding alcohol dehydrogenase (ABAD) [150,154]. Recent studies have also demonstrated that metabolic alterations in the retina of an AD mouse model, including decreased glutamine synthesis and glucose hypometabolism, provide insight into potential neuroprotective pathways [19].

### 3.2. Oxidative Stress and Mitochondrial Dysfunction

Oxidative stress and impaired Ca^2+^ homeostasis contribute to the pathogenesis and progression of AD [119,155]. Upregulation of oxidative phosphorylation (OXPHOS) activity can lead to an increase in ROS that directly affects neurons [6] and glia [112] in the AD brain.

Neurons are fundamentally dependent on mitochondrial OXPHOS to meet their energy needs. When neurons are damaged, they respond by upregulating OXPHOS, better known as the inverse Warburg effect [6]. It shifts from a healthy state to pathological aging with subsequent amyloid plaque accumulation, DNA damage, increased ROS production, and neuronal loss due to their high susceptibility to free radicals [113]. The inverse Warburg effect suggests that an imbalance between the fusion and fission of intact and damaged mitochondria may play an important role in the pathogenesis of AD [6,156]. In AD brain neurons, altered Ca^2+^ influx in dysfunctional mitochondria can lead to altered regulation of neurotransmitters, neurogenesis, neuronal plasticity, and lipid synthesis [157]. It has also been suggested that Aβ enters the mitochondria and interacts with mitochondrial proteins that produce ROS and free radicals [145].

Research has shown that at least 42 metabolic proteins, including glyceraldehyde-3-phosphate dehydrogenase (GAPDH), are downregulated in the AD brain. GAPDH is involved in glucose metabolism and has been shown to interact with amyloid precursor protein (APP). The non-glycolytic activity of GAPDH can be modulated by ROS and GAPDH activity is reduced in the AD brain, which may contribute to the loss of neuronal function and subsequent neurodegeneration [158,159].

It has been suggested that metabolic pathways become dysregulated with age, leading to mitochondrial dysfunction and reduced ATP production [160]. Mitochondrial changes in AD have also been shown to correlate with reduced energy metabolism and oxidative stress [150]. In this context, it is proposed that damaged neurons in an AD brain attempt to protect themselves and compensate for energy deprivation by switching to glycolysis with lactate and pyruvate to promote rapid ATP production. Residual lactate and pyruvate enter the mitochondria and the tricarboxylic acid (TCA) cycle for OXPHOS [161]. Competition for lactate also occurs between neurons, and neurons with upregulated OXPHOS outperform the rest. They have the advantage of using lactate from astrocytic glycolysis as an energy substrate, thus sparing glucose for healthy neurons [160,162]. In addition, a defect in cytochrome c oxidase has been implicated in the bioenergetic deficit of AD [127] as a result of mitochondrial DNA (mtDNA) mutations [163]. In aged animals, reduced glutathione levels have been associated with mitochondrial damage [87,105].

**Figure 4:**
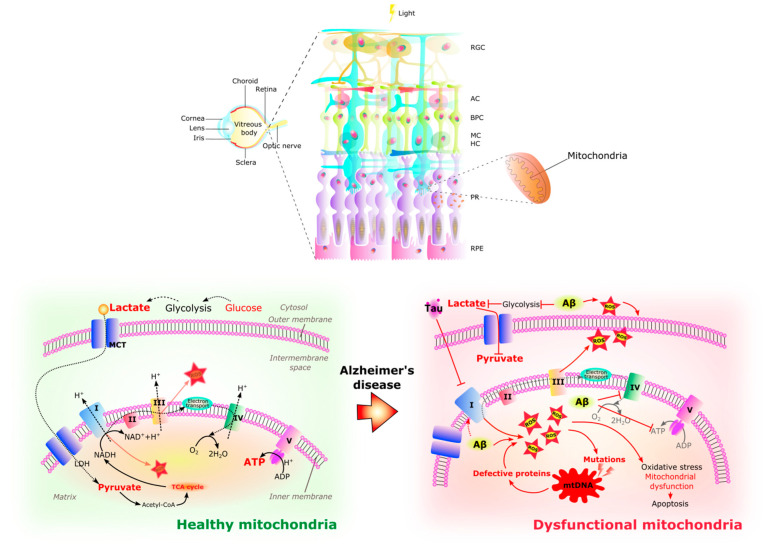
Healthy and dysfunctional mitochondria in the retina. The RPE/photoreceptor complex resides in a highly oxidative environment. A large number of mitochondria are located in the outer end of Müller glia, in the inner segments of photoreceptors, and in the basal part of RPE cells. The retina is a highly energy demanding organ. In healthy retinal cells, glycolysis occurs in the cytoplasm, providing pyruvate and lactate to the mitochondria where they can be substrates for acetyl-CoA synthesis, fueling the TCA cycle and electron transport chain (complex I–V) activity with final energy production in the form of ATP. In the Alzheimer’s disease retina, glycolysis is affected by the amyloid-β (Aβ) peptide causing the increase in reactive oxidative species (ROS), while hyperphosphorylated tau (pTau) inhibits the electron transport chain, provoking the increase of ROS inside the matrix of the mitochondria. In a harmful cycle, the increase in ROS causes mutations in mitochondrial DNA that will produce defective proteins which in turn decreases ATP and increases ROS with the subsequent oxidative stress, mitochondrial damage, and apoptosis which contribute to the retinal pathology of AD. RGC Retinal ganglion cells. AC Amacrine cells. BC Bipolar cells. MC Müller cells. HC Horizontal cells. PR Photoreceptors. RPE Retinal pigmented epithelium. I NADH reductase. II Succinate dehydrogenase. III Cytochrome complex. IV Cytochrome C oxidase. V ATP synthase. TCA cycle Tricarboxylic acid cycle. MCT Monocarboxylate transporter. ROS Reactive oxygen species. mtDNA Human mitochondrial DNA. ATP Adenosine triphosphate. ADP Adenosine diphosphate.

Similar to the brain, the chronic increase of ROS in the AD retina contributes to oxidative stress, mitochondrial dysfunction, and cell death [126,128] (Figure 4). This excessive ROS production is thought to be related to abnormal stimulation of NMDAR by Aβ oligomers [134] and mitochondrial dysfunction. Aβ aggregates bind redox-active metals such as iron and copper, sources of ROS, causing mitochondrial damage and subsequent neurotoxicity [122]. Aβ deposits and NFTs activate retinal astrocytes and microglia [164] with the production of inflammatory cytokines such as IL-1β, IL-6, and TNF-α [165], which together with ROS may cause neurotoxicity. RGCs appear to be particularly susceptible to this neurotoxic effect and thinning of the retinal nerve fiber layer is an increasingly recognized phenomenon in AD [122].

Studies of the retina in mouse models of AD suggest that Aβ peptides are responsible for increased Ca^2+^ in mitochondria, leading to mitochondrial fragmentation, disruption of the fission-fusion balance, and mitochondrial dysfunction [102].

It is still unknown whether mitochondria play a primary or secondary role in the pathogenesis of AD, as it is debated as to whether this is a consequence of amyloid deposition, or whether it is directly involved in the early stages of AD. Therefore, an alternative hypothesis is that amyloid/tau pathology and mitochondrial dysfunction are correlated [166].

## 4. Similarities between Retinal Manifestations in Retinas from AD Patients and Retinal Manifestations in Other Eye Diseases

Studies of retinal diseases are therefore crucial in recognizing the potential of the retina as a window to the brain, providing new insights into neurodegenerative processes.

Common mechanisms and biomarkers of neuronal dysfunction can likely be identified by studying degeneration patterns, proteins, metabolic and energy pathways, and inflammatory responses in different retinal diseases. This approach will not only aid in the early diagnosis of AD but may also potentially improve the understanding of disease progression and the effects of therapeutic interventions (Table 2).

### 4.1. Diabetic Retinopathy and AD

Diabetic retinopathy (DR) is a leading cause of vision loss and is expected to affect more than 190 million people by 2030 [186]. The increasing prevalence of DR is linked to the rise in cases of diabetes associated with the increasing prevalence of obesity and sedentary lifestyles [187]. Altered metabolic parameters and modifiable risk factors predisposing to type 2 diabetes mellitus (T2DM) have been associated with the risk of AD [128,188,189,190,191,192,193]. Similarly, both diabetes and diabetic retinopathy are associated with an increased risk of developing AD [194,195,196] and cognitive impairment [197,198,199,200].

Disruption of insulin signaling and abnormal activation of components of the insulin signaling pathway in the brain, features typical of diabetes, have been reported in AD [128,201,202,203]. The brain has a high density of insulin receptors [204,205], and some studies show that Aβ oligomers are involved in removing insulin receptors (IRs), thereby reducing the IR protein tyrosine kinase activity through TNF-α/c-Jun NH_2_-terminal kinase (JNK) activation [128,206]. In addition, the retina is also an insulin-sensitive tissue, and it has also been suggested that Müller cells may secrete insulin [207] and that a deficiency in receptor signaling may contribute to retinal cell death [208].

Intranasal insulin has been shown to improve memory in healthy adults, verbal memory in patients with memory impairment, and cognitive performance in patients with early AD and mild cognitive impairment [209,210,211]. Insulin has been shown to block the downregulation of IR, IR substrate-1pSer (IRS-1pSer), and oxidative stress, and to protect neurons against synapse loss induced by Aβ oligomers [128,212]. GLP-1R agonists activate insulin signaling pathways through G-protein-dependent signaling. This influences the regulation of glucose metabolism and impairs neurological and cognitive function [213,214]. In GLP-1R agonist-treated mice, reduction of Aβ plaques and microglial activation have been observed, thereby conferring neuroprotection [213].

### 4.2. Glaucoma and AD

Glaucoma is a progressive optic neuropathy, characterized by RGC loss and progressive visual field loss. It is one of the leading causes of blindness worldwide [33,215,216]. As is the case in AD, mitochondrial dysfunction, altered OXPHOS, and increased ROS have been reported to play a role in the pathogenesis of glaucoma [217,218]. In addition, similarities between AD and glaucoma have long been recognized, as the retinal neurodegeneration in AD appears to predominantly affect RGCs [139].

There is evidence that primary open-angle glaucoma (POAG) and AD may share genetic risk factors. For example, the optineurin (OPTN) gene encodes for the optineurin protein [219], which is expressed in the brain, retina, heart, placenta, kidney, and other tissues [220]. Overexpression of OPTN is strongly associated with TNF-α induced death of rat RGC-5 cells [221], suggesting an upregulation in AD [222]. Optineurin neurotoxicity may be a common risk factor in normal tension glaucoma (NTG) and AD [220].

Despite these pathogenic similarities, the association between AD and primary POAG remains controversial [219,223,224]. Some studies have found no association between POAG and AD [225,226], including a meta-analysis in 2021 that found no association between glaucoma and AD (risk ratio (RR): 1.03, 95% confidence interval (CI): 0.93–1.05; I^2^ = 83%, *p*= 0.55) [227]. On the other hand, some studies suggest an association between the two diseases [228,229], including a systematic review and meta-analysis in 2019 that showed an increased risk of AD in glaucoma patients (RR = 1.52; 95% CI: 1.41–1.63; I^2^ = 97%, *p* < 0.001) and an Asian study that found a significant association (RR = 2.03; 95% CI: 1.02–4.07) [230]. In addition, studies in Taiwan, Korea, and Canada have all shown that patients with POAG have a higher risk of developing AD [231,232,233], suggesting that POAG may be a predictor of AD in certain demographic groups. The contradictory evidence of associations between AD and glaucoma may be due to the diagnostic criteria used in each study, including diagnostic misclassification. In addition, the fact that some studies may have included only patients with severe dementia, the small size of the cohort in some studies, the inclusion of case-control studies, which may lead to a positive patient selection bias, and the different mean ages of the study participants may also explain the inconsistent conclusions. Future longitudinal studies are needed to better understand the associations between AD and glaucoma.

### 4.3. Age-Related Macular Degeneration and AD

Age-related macular degeneration (AMD) is another major cause of blindness in people over the age of 50 years [234]. AMD is a chronic degenerative disease of the macula that affects central vision, and it is characterized by the accumulation of drusen between the RPE and Bruch’s membrane [235].

Extracellular deposits of Aβ [236], iron accumulation [237], chronic inflammation, and oxidative stress [122] are common features in the retina of patients with AMD. This suggests a strong correlation and probably overlap in pathology between AD and AMD [15,122,238,239]. Li-Yen Wen et al. have shown a 1.23-fold increased risk in AD patients with AMD [240].

Based on the overlapping features of the diseases, iron chelating agents have been proposed as a potential strategy for the treatment of both AD and AMD [122,241,242], together with therapeutic elimination of Aβ accumulation with anti-Aβ antibodies or reduction of Aβ production with β-secretase inhibitors based on the overlapping mechanisms [138]. Trials of these therapeutic approaches are currently ongoing.

Overall, as mentioned above, some studies have shown an association between AMD and the risk of AD. However, a UK study of 2088 individuals aged 69 to 97 found a lack of a significant association between AD and early AMD, although AD and AMD have similar pathologies, ApoE status, and vascular risk factors. However, they also mention that although the study was large, only 145 of the 707 participants with dementia were considered to have AMD. Also, some of the people with AMD and AD may have died before the study was conducted. In addition, only one eye was examined in the retinal photography study. Finally, the study was a cross-sectional study which meant that it was impossible to know when the cognitive decline occurred in AMD [243].

## 5. Non-Invasive Retinal Imaging to Detect Features of AD

### 5.1. Optical Coherence Tomography (OCT)

The detection of Aβ and tau accumulation in the brain currently relies on the use of PET imaging or CSF analysis, both of which are expensive and invasive [244]. Assuming that AD can be predicted via the retina, it is relevant to explore methods for mapping retinal biomarkers [59]. In this context, OCT is a non-invasive technique that uses low-coherence interferometry to obtain cross-sectional three-dimensional images of the retina, allowing for the study of retinal thinning, macular thinning, and vascular changes [244].

The thickness of the macula as examined by OCT is a valuable indicator of AD. Studies have shown reduced macular volume and thickness in asymptomatic individuals with a high genetic risk of developing AD [245] and reduced central macular thickness in patients with AD compared to healthy controls [246].

In patients with AD, macular and peripapillary RNFL thinning has been reported in numerous studies using OCT [123,247], suggesting that OCT may be used to detect biomarkers for AD [122,239,248,249,250,251]. At present, these changes lack specificity for disease detection, as there is considerable overlap between OCT findings in AD and several other neurodegenerative diseases, including glaucoma. It should be noted, however, that some factors may affect the OCT measurements, such as elevated intraocular pressure, variable axial length, refractive error, systemic comorbidities, or other eye diseases due to similarities in the pathology [252].

**Figure 5:**
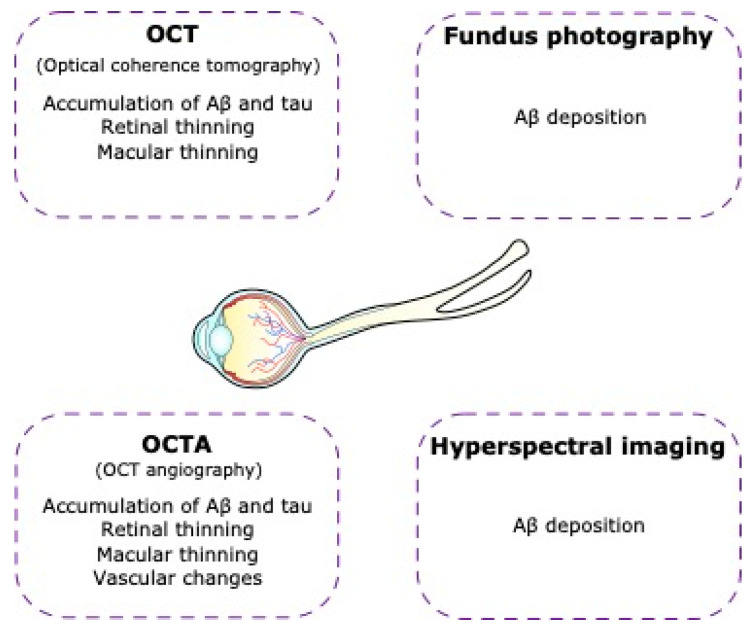
Non-invasive imaging of the retina in Alzheimer’s disease. In Alzheimer’s disease patients there are visual alterations that can lead us to Alzheimer’s disease diagnosis using technological tools such as optical coherence tomography (OCT), optical coherence tomography angiography (OCTA), fundus photography, and hyperspectral imaging.

Nevertheless, the use of OCT is a useful tool in the diagnosis and monitoring of retinal diseases and may therefore be a valuable resource in the diagnosis of neurodegenerative diseases, such as AD [123,253,254,255,256] (Figure 5).

### 5.2. Optical Coherence Tomography Angiography (OCT-A)

Vascular changes in the brain, including cerebral amyloid angiopathy and blood-brain barrier dysfunction, are known to be associated with AD [257]. Similarly, AD has also been associated with vascular changes in the retina [252]. OCT-A provides a detailed non-invasive examination of the retinal vasculature without the use of contrast agents [258]. Studies have shown retinal microvascular network changes, with an enlargement of the FAZ, microvascular density lesions in the deep retinal capillary plexuses, and lower vascular density in whole macular, foveal, and parafoveal zones in people with AD compared to controls [259,260].

Thus, OCT-A may help us detect these changes in the retinal microvasculature before the clinical symptoms of AD appear, which may aid in predicting and evaluating the progression of the disease.

Errors in data acquisition or image quality, as well as ocular anatomy and diseases such as hypertension or diabetes, can affect OCT-A readings [261,262]. As with OCT, other eye diseases can also affect the readings [262]. Furthermore, it must be considered that OCT and OCT-A values may be altered by race, age, or gender [263].

### 5.3. Hyperspectral Imaging

The presence of Aβ deposits in the inner retinal layers of patients with AD has been reported in several studies [264,265]. Hyperspectral imaging is an imaging technique that acquires a series of images across many contiguous wavelengths of light to combine spectral and spatial information into a single data cube [266]. This emerging imaging technique has shown promise in distinguishing AD mice from wild-type mice [264,267,268]. Furthermore, recent studies have shown that hyperspectral imaging may be able to discriminate patients with AD pathology from healthy controls [264,265]. Larger replication studies are needed before it is clear whether this imaging technique has clinical value in AD.

### 5.4. Fundus Photography

Fundus photography is a non-invasive imaging technique that produces two-dimensional color images of the back of the eye, including the retina. This provides detailed images with high sensitivity and specificity. These include vessel caliber, tortuosity, and the global geometric branching network [269]. The microvascular changes in the retinas of patients with AD include narrower retinal venules and more tortuous retinal vessels, which may reflect changes in vascular structure and function that are associated with the brain pathology of AD and dementia [270,271,272].

Although not a standalone diagnostic tool, fundus photography contributes to our understanding of retinal changes in AD and may complement other diagnostic and follow-up approaches in AD.

## 6. Other Approaches to Detect Biomarkers of AD in the Eye

### 6.1. Aβ and pTau in the Vitreous Humor

Reduced levels of Aβ and pTau have been reported in the vitreous humor and correlated with poor cognitive function. Therefore, whilst it may be technically feasible to use the presence of these proteins as a biomarker for AD [273,274], the method’s applicability is limited as it requires surgery to collect intraocular fluid.

### 6.2. Fluorescent Signal of Ligand Bound to Aβ in the Lens

A combination of a fluorescent ligand with an affinity for Aβ and a laser scanning device have been proposed as a means to detect the accumulation of Aβ in the crystalline lens of the eye following application as an eyedrop [275,276], with significant correlations between eye and brain measurements [277]. Further studies of this imaging method are in progress for the detection of AD.

### 6.3. Corneal Confocal Microscopy (CCM)

Corneal confocal microscopy (CCM) is a non-invasive ophthalmic imaging technique that has shown promise in the detection of neuronal loss in neurodegenerative diseases. It can detect changes in corneal neurons and is useful in detecting neuropathies such as diabetic neuropathy [278] and can also identify corneal nerve changes in Parkinson’s disease [279] and multiple sclerosis [280]. The neurotransmitter acetylcholine (ACh) has been shown to play an important role in maintaining the corneal epithelium. Furthermore, ACh is deficient in AD, so changes in corneal structure could be used for the detection and monitoring of AD. Therefore, by analyzing the micromorphology of the corneal subbasal nerve plexus (SNP), CCM may provide insight into peripheral and central neurodegeneration in AD [281].

### 6.4. Ocular Manifestations

Anatomic changes in AD extend beyond the retinal layers to include changes in the size of the pupil and optic nerve. Pupillary abnormalities have been observed in individuals with AD, often manifesting as varying responses of the pupil to constrict and dilate in response to light stimulation [282]. This may be indicative of underlying neurological dysfunction, as the pupillary reflex is influenced by the sympathetic (adrenergic) and parasympathetic (cholinergic) autonomic nervous systems [283], memory function [284], and brain control of the pupillary muscles [283]. To detect these changes in the pupil, pupillometry provides a non-invasive method and a potential biomarker for AD [285].

Another important site of anatomical change in AD is the optic nerve, which connects the eye to the brain. Although not directly observable with routine retinal imaging, there is evidence of retinal ganglion cell degeneration and a widespread axonal degeneration in the optic nerve [286], nerve fiber damage, increased cup-to-disc ratio [287], and with the use of a retinograph, detection of papillary paleness in the optic disk in AD [288]. These changes may be the result of axonal and neuronal degeneration in the optic nerve head [39].

## 7. Conclusions

AD is a neurodegenerative disorder characterized by the abnormal accumulation of Aβ peptides and tau protein in the brain. Several studies have shown that AD patients have abnormal accumulations of both Aβ and tau in the RGC layer and the inner retina, suggesting a possible link between retinal pathology and AD. Accumulating research suggests that these pathological changes occur in the retina over a similar timeframe as in the brain, making the retina an attractive approach to AD diagnosis.

In AD, mitochondrial dysfunction disrupts energy metabolism, leading to impaired cellular function and an increased susceptibility to neurodegeneration. This impairment extends to retinal cells and may contribute to the retinal degeneration seen in AD. Another consequence of mitochondrial dysfunction is an increase in the production of ROS. The accumulation of ROS in the retina can lead to oxidative stress and further exacerbate retinal degeneration. Taken together, retinal Aβ and tau accumulation, along with reduced mitochondrial function, impaired energy metabolism, and increased ROS production, are likely to contribute to retinal degeneration in AD.

Retinal tests, such as OCT and hyperspectral imaging, offer several advantages in detecting AD compared to traditional clinical brain diagnostic methods. Retinal assays are non-invasive, low-cost, time-efficient, and well-tolerated by patients, making them accessible tools for early detection and disease monitoring. They can detect subtle retinal changes, even before the onset of cognitive symptoms in AD.

It is important to note that there are differences between retinal imaging techniques, such as OCT or OCT-A, which provide more detailed information on retinal structure, thickness, and microvascular changes compared to traditional fundus photography or hyperspectral imaging. However, the combination of some techniques, such as hyperspectral imaging and OCT, may lead to a better and faster diagnosis of AD [289]. Other ocular methods, such as CCM, provide insight into the corneal nerve morphology related to AD but do not offer insight into the retina. On the other hand, detection of Aβ by fluorescent signaling in the lens may not provide a complete picture of AD-related changes, and detection of Aβ and pTau in the vitreous humor is an invasive tool requiring surgery.

There are many factors to consider when comparing the cost of retinal techniques for the detection of AD. Methods such as fundus photography are in general more affordable, which makes them more accessible for routine screening. The integration of artificial intelligence (AI) algorithms into these techniques may increase their cost but bring significant benefits such as accuracy and precision. AI can analyze retinal data more comprehensively, potentially leading to the detection of subtle changes associated with early AD. As a result, early detection of AD, early intervention, and increased treatment efficacy would justify this investment.

While retinal and ocular tests are promising, at present they are not standalone diagnostic tools but rather complements to existing clinical techniques. Integration of retinal evaluation with MRI, PET, and other biomarker-based diagnostic approaches may improve the sensitivity and specificity of AD detection, providing a diagnostic strategy that incorporates both brain and retinal pathology.

An understanding of the mechanisms in Alzheimer’s retinas could provide valuable insights into the pathophysiology of AD and potentially facilitate the development of novel approaches for early diagnosis and therapeutic strategies for the disease. The U.S. Food and Drug Administration recently approved two anti-amyloid antibodies (Lecanemab and Aducanumab) as disease-modifying therapies for AD. With this treatment, it stands to reason that the implementation of retinal screening for early diagnosis could serve to initiate treatment at a stage when the damage has not yet caused cognitive impairment in the brain, thus providing neuroprotection.

In summary, there is compelling evidence to support retinal changes as biomarkers for the diagnosis and potential prediction of AD. However, there is a lack of consensus on how to implement such a strategy in clinical practice and further research is needed to improve the understanding of the relationship between retinal manifestations and AD.

## Figures and Tables

**Table 1:** Alzheimer’s-related findings in both brain and retina of human and animal models.

		Findings	In Vitro	In Vivo	Reference
Mouse	Retina	Aβ plaques	X	X	[60,97,98]
		pTau, tau accumulation, and NFTs	X		[99,100,101]
		RGC loss/RNFL thinning	X	X	[60,61,102,103]
		Energetic, metabolic, and mitochondrial dysfunction	X		[19,33,89,104,105,106]
		Microglia activation	X		[15,33,107]
		Müller glia activation	X		[33,97,105]
	Brain	Aβ plaques	X		[103,108]
		pTau, tau accumulation, and NFTs	X		[13,109,110,111]
		Energetic, metabolic, and mitochondrial dysfunction	X		[106,112,113,114]
		Microglia activation	X	X	[12,13,79,93,114,115,116,117]
		Increased oxidative stress	X	X	[112,118,119]
Human	Retina	Aβ plaques	X	X	[29,30,31,38,63,66]
		pTau and NFTs	X	X	[29,38,70]
		Visual impairment	X	X	[45,120]
		RGC loss/RNFL thinning	X	X	[45,59,121,122,123]
		Energetic, metabolic, and mitochondrial dysfunction	X		[104]
		Microglia activation	X		[15,18,33,124]
		Müller glia activation	X		[15,95]
		Increased oxidative stress	X		[15,122]
	Brain	Aβ plaques	X	X	[4,5,117,125]
		pTau and NFTs	X	X	[4,5,125,126]
		Energetic, metabolic, and mitochondrial dysfunction	X	X	[6,118,126,127,128,129,130,131]
		Microglia and neuron activation	X		[12,18,112,117,132,133]
		Increased oxidative stress	X	X	[17,113,134,135]

**Table 2:** Comparison of Alzheimer’s disease with Diabetic Retinopathy, Glaucoma, and Age-Related Macular Degeneration.

	Alzheimer’s Disease	Diabetic Retinopathy	Glaucoma	Age-Related Macular Degeneration
Neurodegeneration	Present	Yes [167]	Present	Yes [168]
Vascularization	Yes	Yes	Yes	Yes
Vision Impairment	Progressive decline	Vision loss	Peripheral vision loss	Central vision loss
Risk Factors	Age, genetics	Diabetes, hypertension, obesity	Age, family history	Age, genetics
Proteins and pathways				
Aβ	Yes [169]	Possible [170]	Yes [171]	Yes [172]
Tau protein	Yes [169]	Possible [173]	Possible [174]	No
Inflammatory pathways	Yes [18]	Yes [175]	Yes [18]	Yes [176]
Vascular dysfunction	Yes [63]	Yes [177]	Yes [178]	Possible [179]
Oxidative stress	Yes [155]	Yes [180]	Yes [181]	Yes [182]
Neuroinflammation	Yes [63]	Yes [183]	Yes [122]	Yes [122]
VEGF	No findings	Yes [184]	Yes [178]	Yes [185]
Treatment	Symptomatic care, no cure, acetylcholinesterase (AChE) inhibitors, memantine, Lecanemab, and Aducanumab	Strict blood sugar control, laser therapy, anti-VEGF therapy, surgery	Medications, surgery	Anti-VEGF therapy, supplements
